# Fourier Shell Analysis: k‐Space‐Based Metrics for Assessing Super‐Resolution in 4D Flow MRI

**DOI:** 10.1002/mrm.70478

**Published:** 2026-06-11

**Authors:** Luuk Jacobs, Pietro Dirix, Sebastian Kozerke

**Affiliations:** ^1^ Institute for Biomedical Engineering, University and ETH Zurich Zurich Switzerland

**Keywords:** 4D flow MRI, computational fluid dynamics, deep learning, image synthesis, open‐source, reproducibility, super‐resolution

## Abstract

**Purpose:**

To support the emerging field of super‐resolution (SR) in 4D flow MRI by proposing Fourier shell analysis to disentangle resolution enhancement from denoising effects during evaluation.

**Methods:**

A thoracic aortic 4D flow MRI dataset was synthesized with various degrees of stenosis, providing ground truth flow fields generated using computational fluid dynamics and MRI‐specific downsampling to generate paired low‐ and high resolution (LR/HR) 4D Flow MRI data. To address potential confounding of resolution enhancement versus denoising effects when using current image‐based error metrics, a k‐space‐based Fourier shell analysis is proposed to compare SR results to HR references using multiple concentric shells with increasing spatial frequency radii in k‐space. To demonstrate its value, the performance of a deep learning SR network (4DFlowNet) with two degrees of transfer learning is compared for three different signal‐to‐noise ratios (SNR) of ∞, 20, and 5.

**Results:**

It is demonstrated that Fourier shell analysis is superior in isolating and quantifying resolution enhancement compared to image‐based metrics. When comparing SR performance of 4DFlowNet with different degrees of transfer learning, Fourier shell analysis isolates resolution enhancement more clearly relative to image‐based normalized root‐mean‐square‐error analysis (absolute relative mean paired difference of 160.3% vs. 13.1% for noiseless data, 92.3% vs. 12.4% for SNR of 20, and 300.0% vs. 10.2% for SNR of 5, respectively).

**Conclusion:**

Fourier shell analysis allows for disentangling and quantifying resolution gain and denoising of SR methods in 4D flow MRI and should serve as an additional metric when assessing SR approaches.

## 1 Introduction

4D flow MRI [1] facilitates quantification of 3D velocity vector fields resolved over the cardiac cycle, from which hemodynamic parameters can be inferred. However, the accuracy (particularly for near‐wall estimations such as wall shear stress) is negatively affected by low signal‐to‐noise and velocity‐to‐noise ratios (SNR/VNR) and the limited spatial resolution [2]. Besides deep learning (DL)‐based image reconstruction methods for improved recovery of high frequency details [3], DL has also been deployed to perform post‐acquisition super‐resolution (SR) and denoising for improved velocity quantification and subsequent hemodynamic parameters inference. Typically, synthetic 4D flow MRI data is used to learn such a mapping from noisy low resolution (LR) to noiseless high resolution (HR) velocity vector fields and for subsequent evaluation. Specifically, geometries and boundary conditions are either generated or extracted from in vivo 4D flow MRI data and “ground truth” velocity vector fields are simulated at theoretically unlimited spatiotemporal resolution using computational fluid dynamics (CFD) [4]. Synthetic paired LR/HR data is then generated via “MRI‐specific” downsampling (consisting of cropping in k‐space and addition of noise) of the CFD data to the desired spatiotemporal resolutions and SNR/VNR levels, which can be used for training and evaluation of SR networks [4].

The SR task can be formulated as an inverse problem that reconstructs HR data from LR inputs, effectively extrapolating k‐space beyond the LR acquisition bandwidth. Following the information capacity theorem of Shannon's information theory [5], stating that the amount of information is limited by the acquisition bandwidth and SNR, new information must be added during SR. Given the increasing availability of realistic synthetic “ground truth” data, most recent works leverage supervised deep learning strategies, broadly including two approaches: (1) Physics‐informed neural networks (PINNs), which add information through physical regularization derived from fluid mechanics relying on time‐consuming iterative approximation of the Navier–Stokes equations for each anatomy and (2) Fully data‐driven neural networks, which offer faster inference by estimating missing high‐frequency information from statistical priors learned from redundancies and structural correlations in the training data. Currently, the latter is more widely adopted, mostly using patch‐based convolutional neural networks (CNN) [6], given their straightforward implementation and optimization, as well as lower computational demands compared to PINNs.

In the field of nonmedical SR of natural images, the importance of evaluation metrics is well regarded, with extensive research on the development of metrics that attempt to mimic human perceptual preferences [7, 8]. Comparatively, the evaluation of 4D flow MRI SR is less complex since: (1) its quantitative nature does not require an analysis of the ambiguity of human perceptual preferences, (2) the increasing availability of paired “ground truth” and test data generated using realistic data synthesis approaches avoids dealing with low quality reference data [9] or even with referenceless evaluations [10], and (3) the well‐defined resolution degradation model in MRI, consisting of low‐pass filtering in k‐space, avoids considering a large degradation space that would arise from more complex and high‐order degradation models as in real‐world SR [11]. Nevertheless, suitable evaluation metrics to assess SR in 4D flow MRI remain to be defined.

Current CNN‐based SR methods almost exclusively rely on voxel‐wise image‐based metrics (see Table S1). Since these metrics are sensitive to global energy differences and the energy contribution of high‐frequency details recovered by SR is relatively small, they are dominated by the denoising capabilities of an SR method, prohibiting insights into how much, if any, new high‐frequency information has been added. Furthermore, current CNN‐based SR methods have used small numbers of often simplified synthetic data (see Table S1), which may compromise the interpretation of SR gains. In this context, the process of spatial downsampling of the CFD‐generated velocity vector fields from unstructured mesh data to structured grids at the desired spatial resolutions is an often‐overlooked detail that particularly affects boundary voxels and related partial voluming.

In this work, a k‐space‐based Fourier shell analysis approach is proposed to disentangle resolution enhancement from denoising effects, providing insights into SR performance. To compare this new analysis to current image‐based approaches, a large CFD‐enhanced synthetic thoracic aortic 4D flow MRI dataset is generated and made publicly available, including paired HR/LR data for healthy and stenotic cases.

## 2 Methods

### 2.1 Synthetic Data Generation

**FIGURE 1:**
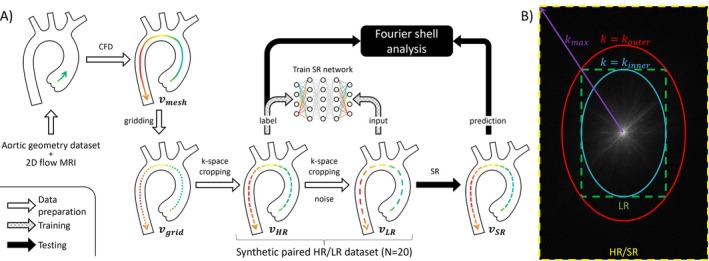
Overview of data generation, SR, and Fourier shell analysis. (A) Pipeline of synthetic data generation, SR, and subsequent analysis. (B) The 3D k‐space with relevant wavenumbers defined as ellipsoids and cuboids are represented in 2D for visualization purposes. LR represents the “acquired” bandwidth and HR/SR the two‐fold increased target bandwidth, defining a pure denoising ($k\leq k_{\text{inner}}$), mixed denoising/SR ($k_{\text{inner}}<k\leq k_{\text{outer}}$), and pure SR ($k>k_{\text{outer}}$) regime.

Twenty 4D flow MRI datasets of the thoracic aorta were synthesized as a subset of the RACLETTE resource [12, 13], containing CFD‐generated ground truth flow fields [13, 14] for an equal number of healthy, mild, moderate, and severe aortic stenosis cases [15, 16]. Geometries were derived from publicly available anatomies [17, 18] and velocity inlet boundary conditions were obtained from 2D flow MRI acquired at the aortic root. The CFD mesh data was temporally averaged to a temporal resolution of 50 ms and projected onto a uniform grid with 0.5 mm isotropic voxel size. Subsequent spatial downsampling was performed by central rectangular boxcar filtering in k‐space to 1.25 mm (HR) and 2.5 mm (LR) isotropic resolution. Three different levels of Gaussian noise were added to the LR k‐space, yielding SNR of ∞ (noiseless), 20, and 5. By first projecting to 0.5 mm isotropic resolution and then downsampling twice to generate HR and LR data, partial volume effects are present in both HR and LR data, resulting in a better posed SR problem compared to if only LR had partial volume effects (Figure 1a).

We note that computing the HR and LR velocity fields directly from the phase of the complex‐valued downsampled image data is methodologically incorrect, given that spatial downsampling of the argument of complex‐valued data is not equal to taking the argument of spatially downsampled complex‐valued data (see Supporting Information). However, to conform with previous approaches described in the literature (see Supporting Information), spatially low‐pass filtered real‐valued velocity fields were also used in this work.

### 2.2 Fourier Shell Analysis

Fourier ring correlation (FRC) [19, 20] and its volumetric extension Fourier shell correlation (FSC) [21] have been used to assess effective image resolution in cryo‐electron microscopy and more recently in optical nanoscopy [22, 23]. Two independent reconstructions of the same object (with independent noise) are Fourier transformed and their normalized cross‐correlation is computed for multiple concentric rings/shells with increasing spatial frequency radii. The effective resolution is then defined by the spatial frequency where the resulting FRC/FSC curve decreases below a pre‐determined threshold [24].

We propose to adapt this concept to a more general Fourier shell analysis to benchmark SR methods in order to disentangle and evaluate the performance of denoising and information gain beyond the acquired bandwidth. This is achieved by computing the coherence (γ, Equation 1) and energy deviation (ED, Equation 2) for $N_{k}$ concentric shells with increasing spatial frequency radii, assessing the directional similarity and magnitude recovery in k‐space with respect to the HR reference data, respectively:

(1) $$\gamma \left(k_{i}\right)=\frac{\sum_{k\in k_{i}}ℱu\left(k\right)\cdot ℱv{\left(k\right)}^{*}}{\sqrt{\sum_{k\in k_{i}}ℱu{\left(k\right)}^{2}\cdot \sum_{k\in k_{i}}ℱv{\left(k\right)}^{2}}+\varepsilon }\;\text{for}\;i\in \left\{1,\ldots ,N_{k}\right\}$$

(2) $$\begin{array}{c} \mathrm{ED}\left(k_{i}\right)=\left|\frac{\sum_{k\in k_{i}}{\left|ℱu\left(k\right)\right|}^{2}}{\sum_{k\in k_{i}}{\left|ℱv\left(k\right)\right|}^{2}+\varepsilon }-1\right|\;\text{for}\;i\in \left\{1,\ldots ,N_{k}\right\} \end{array}$$

with radial wavenumber k, i‐th frequency bin $k_{i}$, SR velocity vector field u, HR velocity vector field v, Fourier transform ℱ, complex conjugate ${◼}^{*}$, and ε=1e−10. The resulting γ and ED curves can be subdivided into a pure denoising regime ($k\leq k_{\text{inner}}$), a mixed denoising and SR regime ($k_{\text{inner}}<k\leq k_{\text{outer}}$), and a pure SR regime ($k>k_{\text{outer}}$) based on the acquisition bandwidth of the LR data (Figure 1b), from which the means can be used as quantitative metrics for denoising (D):

(3) $$\begin{array}{c} D_{p}=\frac{1}{N_{\text{inner}}}\sum_{k_{i}\leq k_{\text{inner}}}p\left(k_{i}\right) \end{array}$$

and SR performance:

(4) $$\begin{array}{c} {\mathrm{SR}}_{p}=\frac{1}{N_{\text{outer}}}\sum_{k_{i}>k_{\text{outer}}}p\left(k_{i}\right) \end{array}$$

with metrics p∈{γ,ED}, where $N_{\text{inner}}$ and $N_{\text{outer}}$ are the number of frequency bins that satisfy $k_{i}\leq k_{\text{inner}}$ and $k_{i}>k_{\text{outer}}$, respectively. Note that this Fourier shell analysis relies on the availability of noiseless HR reference data, making it particularly well‐suited for evaluating SR methods for 4D flow MRI, where such data can be generated using CFD. However, the reliance on noiseless HR reference data does prohibit Fourier shell analysis from being used for evaluating in vivo SR. In this work, metrics are computed over all velocity components and cardiac bins, but these can be investigated independently as well. Moreover, although two‐fold upsampling was investigated in this work, the analysis holds for arbitrary upsampling factors. Finally, the SR task investigated in this work only concerns mapping from LR to HR, not addressing the remaining gap between HR and ground truth velocity vector fields with theoretically infinitely high spatial resolution.

### 2.3 Experiments

Instead of comparing different SR methods, various degrees of transfer learning (TL) were investigated for a single SR method to obtain a controllable increase in denoising and SR performance. To this end, two variants of 4DFlowNet [4] were considered, where the publicly‐available pre‐trained weights were fine‐tuned on our synthetic thoracic aorta 4D flow MRI dataset for 10 (4DFlowNet‐TL10ep) and 100 (4DFlowNet‐TL100ep) epochs. Both of these networks were assessed under three SNR regimes, using LR data with SNR of ∞, 20, and 5 as input. Directly using the pre‐trained weights without any transfer learning yielded poor results and were excluded from the experiments here (see Supporting Information). Training (*N* = 10, resulting in *N* × *N*_frames_ × *N*_patches_ × *N*_augmentations_ = 10 × 20 × 10 × 10 = 20 K samples, or 1 K iterations/epoch for a batch size of 20) and testing (*N* = 10) hyperparameters were all adopted from the original 4DFlowNet implementation [4], apart from the learning rate which was lowered to 2e‐5 for fine‐tuning. Classical upsampling methods, only using LR data with SNR of ∞, as input were included as SR baselines including linear interpolation (LIN) and zero‐padding in k‐space (ZP, including a Hamming filter to reduce Gibbs ringing).

For evaluation, a qualitative comparison of the velocity vector fields and corresponding k‐space magnitudes was performed. Quantitative analysis included the proposed Fourier shell analysis. The image‐based vectorial normalized root‐mean‐square error (${\text{nRMSE}}_{v}$):

(5) $$\begin{array}{c} {\text{nRMSE}}_{v}=\sqrt{\frac{\sum_{i\in M}{\left\|u_{i}-v_{i}\right\|}^{2}}{\sum_{i\in M}{\left\|v_{i}\right\|}^{2}+\varepsilon }} \end{array}$$

and mean directional error (DE):

(6) $$\begin{array}{c} \mathrm{DE}=\frac{1}{\left|M\right|}\sum_{i\in M}1-\frac{\left|u_{i}\cdot v_{i}\right|}{\left\|u_{i}\right\|\left\|v_{i}\right\|+\varepsilon } \end{array}$$

of velocities inside the HR mask M and for near‐wall (edge) velocities, defined as a single‐voxel boundary identified via binary erosion of mask M, and ε=1e−10. Note the analogy between ${\text{nRMSE}}_{v}$ and DE in image space versus ED and γ in k‐space, both probing magnitude information and directional similarity, respectively. Finally, for the same velocities, linear regression analyses were performed to compute the regression slope (k) and coefficient of determination ($R^{2}$).

## 3 Results

**FIGURE 2:**
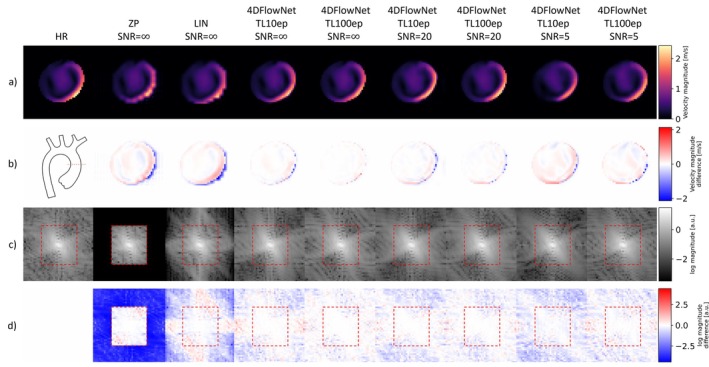
Qualitative comparison of classical upsampling and SR methods. For an exemplary severe stenosis case, the velocity magnitude (a), its differences with HR (b), corresponding k‐space log magnitudes (c), and its differences with HR (d), all scaled and clipped to the same intensities, with the red dashed box defining the bandwidth (BW) are shown for a systolic frame.

The qualitative comparison shows poor recovery of the velocities near the boundaries for the classical upsampling methods, a gradually compromised recovery of bulk flow for decreasing SNR, and an apparent sharpening with the number of fine‐tuning epochs of 4DFlowNet at all three SNR levels (Figure 2).

**FIGURE 3:**
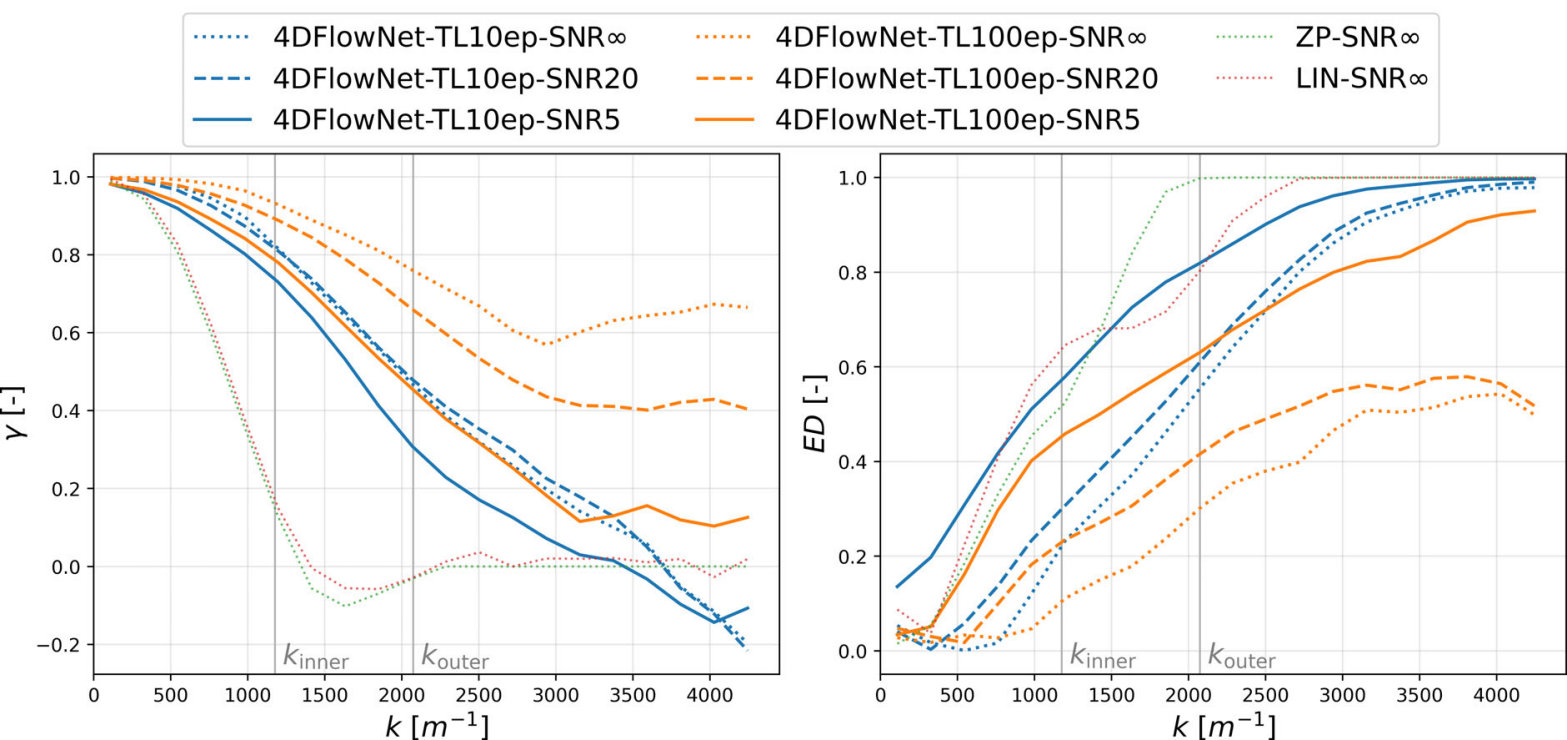
Fourier shell analysis curves comparing classical upsampling and SR methods. For an exemplary severe stenosis case, the coherence (left, γ, ↑) and energy deviation (right, ED, ↓) are given for multiple ($N_{k}=20$) concentric shells with increasing spatial frequency radii. Metrics for methods using LR data with SNR of ∞, 20, and 5 as input are shown using dotted, dashed, and solid lines, respectively.

The corresponding γ and ED curves derived from Fourier shell analysis show a similar pattern, with the classical upsampling performing poorly below and beyond the acquired bandwidth while the 4DFlowNet shows decreasing performance with lower SNR levels and improved performance with increased number of training epochs (Figure 3). Moreover, it can be seen that the SNR level of the LR input affects the performance of 4DFlowNet both below and beyond the acquired bandwidth.

**FIGURE 4:**
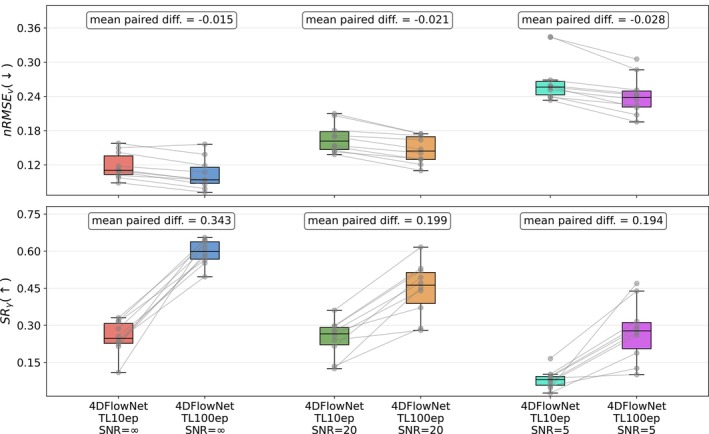
Quantitative comparison of 4DFlowNet with different number of TL epochs. Exemplary image‐based metric (${\text{nRMSE}}_{v}$) (top) and Fourier shell analysis‐based metrics (${\mathrm{SR}}_{\gamma }$) (bottom), using LR data with SNR of ∞ (left), 20 (middle), and 5 (right) as input.

These observed patterns are largely reflected by the quantitative image‐based and Fourier shell analysis metrics, with only LIN outperforming the CNN‐based SR methods for LR data with SNR of ∞ as input in terms of DE near the vessel wall (Table 1). Moreover, it can be seen that most image‐based and all Fourier shell analysis metrics show improvement with more TL. To better illustrate the value of the metrics derived from Fourier shell analysis, the ${\text{nRMSE}}_{v}$ and ${\mathrm{SR}}_{\gamma }$ values are visualized as examples using boxplots (Figure 4), where two observations can be made: (I) ${\mathrm{SR}}_{\gamma }$ is more sensitive to model improvement when using more TL epochs compared to ${\text{nRMSE}}_{v}$ (absolute relative mean paired difference of 160.3% vs. 13.1% for SNR of ∞, 92.3% vs. 12.4% for SNR of 20, and 300.0% vs. 10.2% for SNR of 5, respectively) and (II) while ${\text{nRMSE}}_{v}$ yields an unexpected increase in TL‐based performance gain when adding noise to the LR input data (−0.015 to −0.021 to −0.028, with lower being better), ${\mathrm{SR}}_{\gamma }$ does not show this (0.343 to 0.199 to 0.194, with higher being better). The latter observation demonstrates the oversensitivity of the image‐based ${\text{nRMSE}}_{v}$ metric to denoising performance, given that a better network (TL100ep) is not expected to have a better SR performance gain compared to a worse network (TL10ep) when adding noise to the LR input data; rather it presents a better denoiser. Contrarily, ${\mathrm{SR}}_{\gamma }$ does not show this unexpected behavior thanks to its effective decoupling of SR and denoising performance.

**TABLE 1:** Quantitative comparison of classical upsampling and SR methods.

		Image‐based								Fourier shell analysis			
		${\text{nRMSE}}_{v}$ (↓)	${\text{nRMSE}}_{v}$ (edge) (↓)	DE (↓)	DE (edge) (↓)	k (→1)	k (edge) (→1)	$R^{2}$ (↑)	$R^{2}$ (edge) (↑)	$D_{\gamma }$ (↑)	$D_{\mathrm{ED}}$ (↓)	${\mathrm{SR}}_{\gamma }$ (↑)	${\mathrm{SR}}_{\mathrm{ED}}$ (↓)
SNR = ∞	ZP	0.236±0.078	0.392±0.058	0.0178±0.0104	0.0179±0.0099	1.039±0.030	1.157±0.065	0.937±0.041	0.849±0.064	0.769±0.081	0.246±0.007	−0.002±0.006	1.000±0.000
	LIN	0.206±0.048	0.280±0.036	0.0142±0.0044	0.0165±0.0074	1.048±0.037	1.098±0.054	0.956±0.018	0.922±0.025	0.743±0.066	0.308±0.013	0.037±0.024	0.981±0.013
	4DFlowNet‐TL10ep	0.118±0.022	0.244±0.026	0.0098±0.0022	0.0212±0.0046	1.016±0.011	1.059±0.017	0.985±0.005	0.938±0.012	0.942±0.008	0.091±0.020	0.253±0.062	0.869±0.019
	NL4DFlowNet‐TL100ep	0.104±0.025	0.225±0.036	0.0093±0.0025	0.0266±0.0099	1.012±0.009	1.027±0.022	0.988±0.005	0.945±0.017	0.974±0.006	0.043±0.020	0.596±0.047	0.500±0.026
SNR = 20	4DFlowNet TL10ep	0.167±0.024	0.309±0.056	0.0365±0.0138	0.0741±0.0243	1.000±0.016	1.027±0.042	0.970±0.009	0.894±0.048	0.918±0.017	0.096±0.049	0.248±0.069	0.879±0.017
	4D FlowNet TL100ep	0.146±0.022	0.258±0.068	0.0298±0.0094	0.0633±0.0164	0.994±0.012	0.996±0.032	0.977±0.007	0.924±0.048	0.936±0.020	0.089±0.036	0.447±0.101	0.531±0.050
SNR = 5	4DFlowNet TL10ep	0.269±0.039	0.402±0.058	0.0961±0.034	0.142±0.044	1.042±0.038	1.101±0.118	0.925±0.019	0.837±0.053	0.836±0.034	0.272±0.089	0.081±0.036	0.959±0.009
	4DFlowNet TL100ep	0.241±0.032	0.363±0.070	0.0827±0.026	0.120±0.036	1.000±0.027	1.026±0.087	0.939±0.016	0.856±0.062	0.872±0.032	0.177±0.060	0.275±0.113	0.767±0.037

*Note:* Computed using LR data with SNR of ∞ (top), 20 (bottom), and 5 (bottom) as input, where the best values (mean ± standard deviation) per table, based on the mean, are in bold.

## 4 Discussion

In this work, Fourier shell analysis has been proposed to disentangle SR resolution enhancement from denoising effects. Using a synthetic thoracic aorta 4D flow MRI dataset, it is demonstrated that CNN‐based SR methods can inject meaningful information compared to classical upsampling methods. Moreover, the qualitatively poor performance of the pre‐trained weights compared to the TL networks showcases the variability in synthetic data generation (see Supporting Information), highlighting the need for standardization.

Even though CNN‐based SR performance remains dependent on the SNR of the LR input data, it has been shown here that Fourier shell analysis can isolate resolution gains better than image‐based metrics. Moreover, the definition of an HR mask required for the computation of image‐based metrics can additionally confound results as the mask generated in HR space may not faithfully define the flow domain in SR space given spatially blurring. Note that the proposed Fourier shell analysis does not require masking of the velocity vector fields and hence would appropriately penalize misalignment of boundaries between HR and SR domains, particularly when using metric γ, which probes the angle/phase relationship between k‐spaces and in turn, following the Fourier shift theorem, the alignment of edge locations in image space. Compared to higher‐order metrics as used in evaluation of nonmedical SR, such as Wavelet‐based information fidelity criterion (IFC) [25], Fourier shell analysis offers two benefits: (1) It can be directly linked to the definition of the acquired spatial resolution in MRI via the maximum k‐space bandwidth ($k_{\text{inner}}$ and $k_{\text{outer}}$), as opposed to more empirical definitions of “high” frequencies, and, (2) It offers insights into SR performance by resolving across wavenumbers (quantized by the user‐defined shell width).

Differences in k‐space values within the BW can be seen between HR and ZP at SNR of ∞ (Figure 2), reflected by suboptimal $D_{\gamma }$ and $D_{\mathrm{ED}}$ values for Fourier shell analysis (Figure 3 and Table 1). This difference is a consequence of the synthetic data generation pipeline, following SR methods implementations as described in the literature, which operate on real‐valued velocity vector fields (see Supporting Information). However, this bias affects all classical upsampling and SR methods equally, making it not a problem for relative comparisons. This observation, together with the fact that these metrics are less directly interpretable on their own than classical voxel‐wise measures suggests that they are best suited for relative comparisons between different SR methods rather than as standalone absolute metrics. A remaining limitation is the reliance on noiseless HR reference data, prohibiting the Fourier shell analysis from being used for evaluating in vivo SR performance.

Future work should aim to bridge the gap to in vivo SR by investigating more realistic downsampling by simulating realistic anatomical features around the vessels of interest, which affect signal spilling into the vascular domain due to the imaging point‐spread function. Finally, instead of the simplistic MRI signal model currently used to generate the synthetic dataset, more advanced MRI simulations shall be used to account for encoding and decoding errors caused by different scales of motion during data acquisition [26].

Fourier shell analysis may also be used for evaluating temporal SR methods for 4D flow MRI, such as the recent adaptation of 4DFlowNet that focused on left‐ventricular flow dynamics [27]. To this end, Fourier “shell” analysis would require a 1D Fourier transform along the cardiac dimension and computation of γ and ED values for each bin of temporal frequencies, with the acquired temporal resolution defining the bandwidth beyond which information gain is expected. Moreover, Fourier shell analysis is not limited to SR in 4D flow MRI and can be applied to any domain where noiseless HR data can be generated and the forward downsampling/observation operation is known. For example, it may find value in fluid mechanics [28, 29, 30], micrometeorology [31, 32, 33], seismology [34, 35, 36], and astronomy [37, 38, 39], where the same synthetic paired HR/LR data generation paradigm has been used for training and evaluating DL‐based SR methods.

## 5 Conclusion

In this work, Fourier shell analysis has been proposed and tested to effectively disentangle resolution enhancement from denoising effects in 4D flow MRI super‐resolution.

## Funding

This project was partially supported by grant #2022‐274 of the Strategic Focus Area “Personalized Health and Related Technologies (PHRT)” of the ETH Domain (Swiss Federal Institutes of Technology).

## Supporting information


**Table S1:** Overview of current CNN‐based SR methods for 4D flow MRI. Internal carotid artery (ICA); Cerebral vasculature (CV); Alzheimer's disease (AD); Relative error (RE); Absolute error (AE); Directional error (DE); Peak signal‐to‐noise ratio (PSNR); Peak velocity‐to‐noise ratio (PVNR); Root MSE (RMSE); Normalized RMSE (nRMSE); Mean arctangent absolute percentage error (MAAPE); Structural similarity metric (SSIM); Wall shear stress (WSS).
**Figure S1:** Overview of SR, ZP, and Fourier shell analysis.
**Figure S2:** Qualitative comparison of classical upsampling versus 4DFlowNet using the publicly available pre‐trained (PT) weights (4DFlowNet‐PT) and fine‐tuned weights with 10 (4DFlowNet‐TL10ep) and 100 (4DFlowNet‐TL100ep) training epochs.

## Data Availability

Synthetic 4D flow MRI data with paired high and low resolution data is publicly available at https://gitlab.ethz.ch/ibt‐cmr/publications/raclette along with code for Fourier shell analysis at https://gitlab.ethz.ch/ibt‐cmr/publications/fourier_shell_analysis.
