# Supplementary material for: Fourier Shell Analysis: k‐Space‐Based Metrics for Assessing Super‐Resolution in 4D Flow MRI
Source: Magn Reson Med. 2026 Jun 11;96(4):1983–90. doi: 10.1002/mrm.70478 (PMC13418888; doi:10.1002/mrm.70478)
Supplement: Supplementary file 1 — Table S1: Overview of current CNN‐based SR methods for 4D flow MRI. Internal carotid artery (ICA); Cerebral vasculature (CV); Alzheimer's disease (AD); Relative error (RE); Absolute error (AE); Directional error (DE); Peak signal‐to‐noise ratio (PSNR); Peak velocity‐to‐noise ratio (PVNR); Root MSE (RMSE); Normalized RMSE (nRMSE); Mean arctangent absolute percentage error (MAAPE); Structural similarity metric (SSIM); Wall shear stress (WSS). Figure S1: Overview of SR, ZP, and Fourier shell analysis. Figure S2: Qualitative comparison of classical upsampling versus 4DFlowNet using the publicly available pre‐trained (PT) weights (4DFlowNet‐PT) and fine‐tuned weights with 10 (4DFlowNet‐TL10ep) and 100 (4DFlowNet‐TL100ep) training epochs. [file MRM-96-1983-s001.docx]

Supplementary material “Fourier shell analysis: A k-space-based metric to assess super-resolution in 4D flow MRI”

Luuk Jacobs^1^, Pietro Dirix^1^, Sebastian Kozerke^1^

^1^ Institute for Biomedical Engineering, University and ETH Zurich, Zurich, Switzerland

# Related work

List of current convolutional neural network (CNN)-based SR methods for 4D flow MRI using synthetic computational fluid dynamic (CFD)-generated data for training, specifying the anatomical region of interest and the quantitative metrics used for evaluation (Tab. S1).

**Table S1: Overview of current CNN-based SR methods for 4D flow MRI.** Internal carotid artery (ICA); Cerebral vasculature (CV); Alzheimer’s disease (AD); Relative error (RE); Absolute error (AE); Directional error (DE); Peak signal-to-noise ratio (PSNR); Peak velocity-to-noise ratio (PVNR); Root MSE (RMSE); Normalized RMSE (nRMSE); Mean arctangent absolute percentage error (MAAPE); Structural similarity metric (SSIM); Wall shear stress (WSS).

| **Method** | **Anatomical region (synthetic only)** | **Quantitative evaluation**  **(synthetic only)** |
| --- | --- | --- |
| Ferdian et al. [1]  (4DFlowNet) | Thoracic aorta (N=1 healthy, N=2 coarctation) | - (Cardiac-resolved) velocity magnitude RE [-]  - Bland-Altman analysis of velocity [m/s]  - (Systolic/diastolic) velocity difference [m/s]  - (Cardiac-resolved) flow rate difference [m^3^/s] |
| Shit et al. [2]  (SRflow) | ICA (N=6 aneurysms) | - (Cardiac-resolved) PSNR [dB]  - Velocity magnitude RMSE [m/s]  - Velocity DE [-]  - Root-mean-squared divergence [s^-1^] |
| Long et al. [3] | Cylinder (N=10 constrictions) | - Velocity magnitude MAAPE [-]  - Velocity RMSE [m/s]  - Velocity SSIM [-]  - Linear regression of velocity [-]  - Bland-Altman analysis of velocity [m/s] |
| Ferdian et al. [4]  (4DFlowNet) | Arterial CV (N=1 healthy, N=2 ICA stenoses, N=1 post-op ICA stenosis) | - (Core/edge) linear regression of velocity [-]  - (Core/edge) Bland-Altman analysis of velocity [m/s]  - (Core/edge) peak velocity magnitude RMSE [m/s]  - (Core/edge) peak velocity magnitude cosine similarity [-]  - (Core/edge) peak velocity magnitude RE [-]  - Flow rate RMSE [m^3^/s]  - Flow rate difference [m^3^/s]  - Linear regression of relative pressure [-]  - Bland-Altman analysis of relative pressure [Pa]  - Peak relative pressure RE [-] |
| Ericsson et al. [5] | Left heart (N=4 mitral regurgitation) + Thoracic aorta (N=1 healthy, N=2 coarctation, N=1 dissection) + Arterial CV (N=1 healthy, N=2 ICA stenoses, N=1 post-op ICA stenosis) | - Velocity magnitude RE [-]  - (Fluid/non-fluid) velocity RMSE [m/s]  - Linear regression of velocity components [-] |
| Patel et al. [6]  (div-mDCSRN-Flow) | Cerebral ventricles (N=5 healthy, N=5 AD) | - Cumulative normalized divergence [-]  - (Core/edge) velocity RMSE [m/s] |
| Odeback et al. [7]  (4DFlowGAN) | Arterial CV (N=1 healthy, N=2 ICA stenoses, N=1 post-op ICA stenosis) | - (Core/edge + low/high SNR + full/systole) velocity RE [-]  - (Core/edge + low/high SNR + full/systole) velocity AE [-]  - (Core/edge + low/high SNR + full/systole) velocity nRMSE [-]  - (Core/edge + low/high SNR + full/systole) velocity DE [-]  - (Core/edge + low/high SNR + full/systole) linear regression of velocity components [-] |
| Perrin et al. [8]  (SURFR-Net) | Thoracic aorta (N=1 healthy, N=2 coarctations) | - (Systolic/diastolic) velocity RE [-]  - (Systolic/diastolic) PVNR [dB]  - (Systolic/diastolic) velocity magnitude nRMSE [-]  - (Systolic/diastolic) velocity DE [-] |
| Zheng et al. [9]  (4DFlowNet) | Carotid artery (N=1) | - Mean surface + Hausdorff distance [mm]  - Velocity + vorticity RE [-]  - Velocity + vorticity kurtosis RE [-]  - Velocity + vorticity skewness RE [-]  - Flow rate AE [m^3^/s]  - WSS RE [-] |

# Real-valued super-resolution

A simplified overview of the super-resolution (SR) pipeline, classical upsampling (for example, zero-padding in k-space [ZP]), and Fourier shell analysis used in the main text is shown in Fig. S1, with complex-valued image data $x$ and velocity vector fields $v=arg(x)\frac{VENC}{\pi}$ at high-resolution (HR) and low-resolution (LR), with velocity encoding (VENC) and downsampling ($\mathcal{D}$).


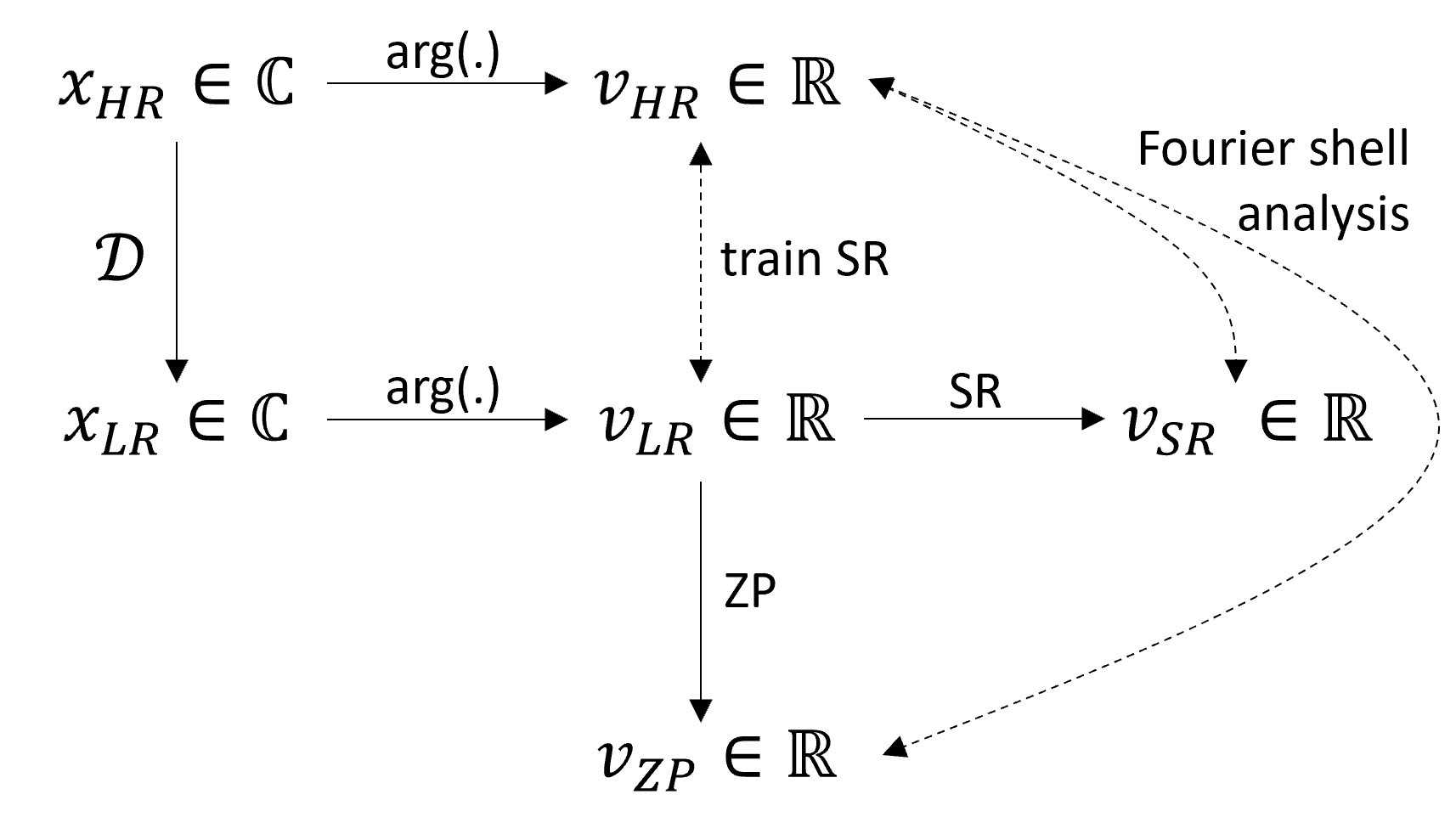


**Figure S1: Overview of SR, ZP, and Fourier shell analysis.**

Here, $\mathcal{D}$ is defined as a central cropping in k-space at bandwidth (BW), here this is an ellipsoidal cropping to simplify notation, but in the main text it is a rectangular cropping. Additionally, no Gaussian noise is added to isolate the problem at hand, such that:

$$\begin{aligned} \mathcal{F\{}x_{HR}\}=\mathcal{F\{}x_{LR}\} \mathrm{for} k<BW,\#\left( 1 \right) \end{aligned}$$

with Fourier transform ℱ, and radial wavenumber $k$. This downsampling must be performed on the image data containing both phase and magnitude components to accurately model partial volume effects. Because current SR methods work almost exclusively with velocity vector fields instead of the complex-valued image data, the phase must be computed using a complex argument operation, which is not commutative with $\mathcal{D}$:

$$\begin{aligned} \mathcal{D}\circ arg(x)\neq arg(\mathcal{D}\circ x).\#\left( 2 \right) \end{aligned}$$

Because we compute the complex argument after the downsampling (Fig. S1), the equality of k-space that one may expect below BW between HR and LR, as imposed by $\mathcal{D}$, does not hold for $v_{HR}$ and $v_{LR}$ and, in turn, also not for $v_{HR}$ and $v_{ZP}$:

$$\begin{aligned} \mathcal{F\{}v_{HR}\}\neq\mathcal{F\{}v_{LR/ZP}\} \mathrm{for} k<BW.\#\left( 3 \right) \end{aligned}$$

This mismatch in k-space centers is not strictly an issue for SR training, which operates in image domain, but does result in “unintuitively” high $nRMSE$ and low $\gamma$ values below the BW for the Fourier shell analysis, which operates in k-space. Note that we could “solve” this by 1) computing the complex argument before the downsampling step i.e. downsampling would be performed on real-valued velocity data, without proper modeling of partial volume effects, or 2) not computing the complex argument at all and performing SR and upsampling on complex-valued data, which, although preferred, would not be compatible with current SR methods described in the literature. Given neither of these options are desirable and the Fourier shell analysis is meant as a relative metric to compare SR methods, we opted to retain this subtlety in the analysis.

# Pre-trained 4DFlowNet

Qualitative comparison of classical upsampling (zero-filling (ZF) and linear interpolation (LIN)), using LR data with SNR=inf as input, with 4DFlowNet SR results based on the publicly available pre-trained (PT) weights (4DFlowNet-PT) and fine-tuned weights using 10 (4DFlowNet-TL10ep) and 100 (4DFlowNet-TL100ep) epochs of transfer learning (TL) (Fig. S2). For clarity, the only the 4DFlowNet results using LR data with SNR=20 as input are not shown here.


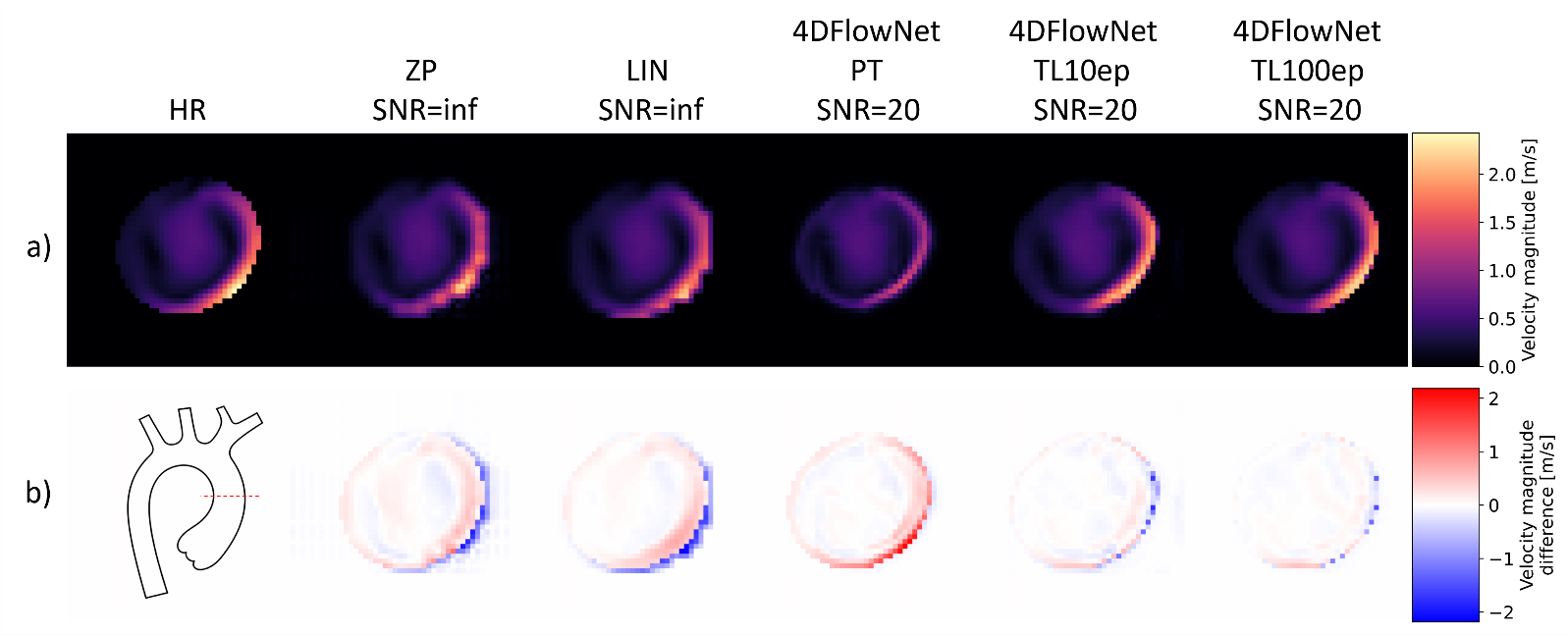


**Figure S2: Qualitative comparison of classical upsampling versus 4DFlowNet using the publicly available pre-trained (PT) weights (4DFlowNet-PT) and fine-tuned weights with 10 (4DFlowNet-TL10ep) and 100 (4DFlowNet-TL100ep) training epochs.**

# References

[1] E. Ferdian *et al.*, “4DFlowNet: Super-Resolution 4D Flow MRI Using Deep Learning and Computational Fluid Dynamics,” *Front. Phys.*, vol. 8, 2020, Accessed: Oct. 14, 2022. [Online]. Available: https://www.frontiersin.org/articles/10.3389/fphy.2020.00138

[2] S. Shit *et al.*, “SRflow: Deep learning based super-resolution of 4D-flow MRI data,” *Front. Artif. Intell.*, vol. 5, 2022, Accessed: Oct. 20, 2022. [Online]. Available: https://www.frontiersin.org/articles/10.3389/frai.2022.928181

[3] D. Long *et al.*, “Super-resolution 4D flow MRI to quantify aortic regurgitation using computational fluid dynamics and deep learning,” *Int. J. Cardiovasc. Imaging*, vol. 39, no. 6, pp. 1189–1202, Jun. 2023, doi: 10.1007/s10554-023-02815-z.

[4] E. Ferdian *et al.*, “Cerebrovascular super-resolution 4D Flow MRI – Sequential combination of resolution enhancement by deep learning and physics-informed image processing to non-invasively quantify intracranial velocity, flow, and relative pressure,” *Med. Image Anal.*, vol. 88, p. 102831, Aug. 2023, doi: 10.1016/j.media.2023.102831.

[5] L. Ericsson *et al.*, “Generalized Super-Resolution 4D Flow MRI - Using Ensemble Learning to Extend Across the Cardiovascular System,” *IEEE J. Biomed. Health Inform.*, vol. 28, no. 12, pp. 7239–7250, Dec. 2024, doi: 10.1109/JBHI.2024.3429291.

[6] N. M. Patel *et al.*, “Super-Resolving and Denoising 4D flow MRI of Neurofluids Using Physics-Guided Neural Networks,” *Ann. Biomed. Eng.*, vol. 53, no. 2, pp. 331–347, Feb. 2025, doi: 10.1007/s10439-024-03606-w.

[7] O. W. Odeback *et al.*, “Potential and challenges of generative adversarial networks for super-resolution in 4D Flow MRI,” Aug. 20, 2025, *arXiv*: arXiv:2508.14950. doi: 10.48550/arXiv.2508.14950.

[8] S. Perrin, S. Levilly, H. Mouchère, and J.-M. Serfaty, “Super-Resolution and Segmentation of 4D Flow MRI Using Deep Learning and Weighted Mean Frequencies,” in *Medical Image Computing and Computer Assisted Intervention – MICCAI 2025*, J. C. Gee, D. C. Alexander, J. Hong, J. E. Iglesias, C. H. Sudre, A. Venkataraman, P. Golland, J. H. Kim, and J. Park, Eds., Cham: Springer Nature Switzerland, 2026, pp. 552–561. doi: 10.1007/978-3-032-04965-0_52.

[9] S. Zheng, A. Mokhtari, B. Jung, A. Harloff, and D. Obrist, “Uncertainty Quantification in Hemodynamic Metrics from 4D Flow MRI with Super-resolution in a Carotid Bifurcation Model,” *J. Imaging Inform. Med.*, Jan. 2026, doi: 10.1007/s10278-025-01796-w.
